# Impact of Nanoscale Morphology on Charge Carrier Delocalization and Mobility in an Organic Semiconductor

**DOI:** 10.1002/adma.202104852

**Published:** 2021-09-23

**Authors:** Matthew Ellis, Hui Yang, Samuele Giannini, Orestis G. Ziogos, Jochen Blumberger

**Affiliations:** ^1^ Department of Physics and Astronomy and Thomas Young Centre London University College London Gower Street London WC1E 6BT UK

**Keywords:** charge transport, crystallinity, non‐adiabatic dynamics, organic semiconductors, quantum delocalization

## Abstract

A central challenge of organic semiconductor research is to make cheap, disordered materials that exhibit high electrical conductivity. Unfortunately, this endeavor is hampered by the poor fundamental understanding of the relationship between molecular packing structure and charge carrier mobility. Here a novel computational methodology is presented that fills this gap. Using a melt‐quench procedure it is shown that amorphous pentacene spontaneously self‐assembles to nanocrystalline structures that, at long quench times, form the characteristic herringbone layer of the single crystal. Quantum dynamical simulations of electron hole transport show a clear correlation between the crystallinity of the sample, the quantum delocalization, and the mobility of the charge carrier. Surprisingly, the long‐held belief that charge carriers form relatively localized polarons in disordered OS is only valid for fully amorphous structures—for nanocrystalline and crystalline samples, significant charge carrier delocalization over several nanometers occurs that underpins their improved conductivities. The good agreement with experimentally available data makes the presented methodology a robust computational tool for the predictive engineering of disordered organic materials.

## Graphical abstract

The hole carrier wavefunction is propagated through samples of pentacene with varying levels of structural disorder using surface hopping non‐adiabatic molecular dynamics. It is found that charge carriers are more delocalized, that is, more “quantum” than previously thought, even for relatively disordered samples, and the mechanism of charge transport as a function of crystallinity is established.

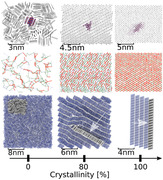

## 1 Introduction

Organic semiconductors (OS) are an exciting class of materials that have enabled disruptive technologies including large area electronics and displays, organic light emitting diodes^[^ ^1^, ^2^ ^]^ and flexible solar cells.^[^ ^3^, ^4^ ^]^ All of these technologies rely on the motion of electrical charges within the OS, commonly quantified by the charge carrier mobility, and efficient device performance is often critically dependent on this important transport coefficient. It has long been recognized that it is primarily the extended solid state structure, in particular the molecular packing and the presence of structural defects that determines, and often limits, the charge mobility than the chemical structure of the molecule or polymer. Indeed, organic field‐effect transistor (OFET) mobilities typically drop by orders of magnitude when going from single crystalline to amorphous samples. As the manufacturing of crystalline samples with low defect concentration is costly and time intensive, the development of conductive disordered materials is a highly desirable goal. Here a fundamental understanding of the relationship between structural disorder and charge mobility is crucial to inform the future engineering of such materials.

Several experimental as well as computational studies have indicated that charge transport in crystalline molecular OS falls into a difficult regime where the charge is neither fully delocalized over the bulk material nor completely localized on a single molecule,^[^ ^5^, ^6^, ^7^ ^]^ as had often been assumed.^[^ ^8^, ^9^, ^10^, ^11^ ^]^ We have recently shown, using advanced quantum dynamical simulations, that charge carriers in single‐crystalline OS form “flickering polarons,” objects that are half‐way between waves and particles.^[^ ^12^, ^13^, ^14^ ^]^ We found they are delocalized over up to 10–20 molecules in the most conductive crystals and constantly change their shape and extension under the influence of the thermal motion of the atoms (crystal vibrations).^[^ ^12^ ^]^ Taking the example of bulk crystalline pentacene, we found that the excess hole is typically delocalized over 17 molecules,^[^ ^12^, ^13^ ^]^ in excellent agreement with experimental estimates from electron spin resonance data.^[^ ^15^ ^]^ The computed mobility, of 9.6 cm^2^ V^−1^ s^−1^, ^[^ ^13^ ^]^ is also in good agreement with experiment, 5.6 cm^2^ V^−1^ s^−1^.^[^ ^16^ ^]^ The delocalization of the polaron, and mobility, are limited by the thermal fluctuations of electronic coupling (off‐diagonal electron–phonon coupling) and site energy (diagonal electron–phonon coupling). This picture, emerging from direct propagation of the time‐dependent electronic Schrödinger equation coupled to nuclear motion, resembles closely, and gives support to, the transport scenario predicted by alternative approaches including transient localization theory (TLT)^[^ ^17^, ^18^ ^]^ and delocalized charge carrier hopping based on generalized Marcus theory^[^ ^19^ ^]^ or polaron‐transformed Redfield theory^[^ ^20^ ^]^ mapped onto kinetic Monte Carlo.^[^ ^21^ ^]^

Here we investigate how structural disorder of the OS, on top of thermal disorder, changes the physical nature of the charge carrier, its localization length, transport mechanism and mobility. In particular, we examine at which degree of structural disorder the flickering polaron loses its delocalized character and becomes localized. This is important because a decrease in charge carrier delocalization correlates with a decrease in charge mobility—the central result of TLT^[^ ^17^, ^18^ ^]^ and of our previous simulations.^[^ ^12^, ^13^, ^14^ ^]^ To do so, we present atomistic quantum dynamical calculations of the charge carrier dynamics at room temperature for a set of pentacene samples with varying levels of crystallinity, from fully amorphous to nanocrystalline to single crystalline. Our quantum dynamical simulation method, denoted fragment orbital‐based surface hopping (FOB‐SH), is well suited for this task because it makes no assumptions with regard to the charge transport mechanism. FOB‐SH was shown to predict charge mobilities well over several orders of magnitude but it has so far only been applied to single‐crystalline OS. Recent methodological developments have now made it possible to apply this novel methodology, for the first time, to large samples of disordered OS with different nanoscale morphologies.

## 2 Results and Discussion

### 2.1 Nanoscale Morphology

**Figure 1:**
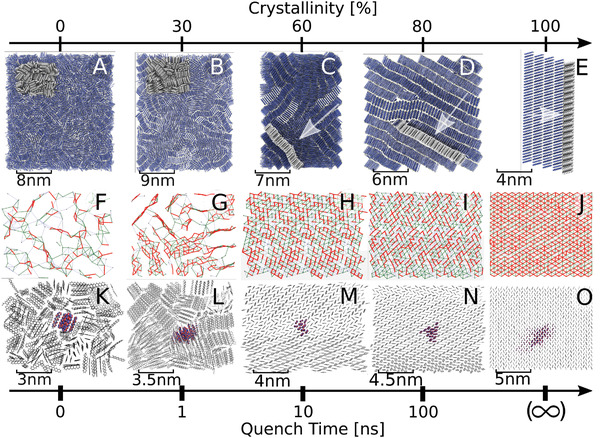
Structure and electronic properties of bulk pentacene phases. The disordered structures were obtained from melt‐quench molecular dynamics simulation. In the upper row, panels A–D show a “front on” view of the entire simulated sample of 3000 molecules for A) amorphous and B–D) nanocrystalline phases. E) Experimental structure of single‐crystalline pentacene (polymorph I).^[^ ^46^ ^]^ The region highlighted in light gray, in the following denoted “active region,” is shown enlarged in the middle and bottom rows and viewed “front on” or as indicated by arrows. The middle row, panels F–J, shows weighted graphs of electronic couplings (calculated with the analytic overlap method^[^ ^47^ ^]^) within the active regions. Molecular centers of mass are joined with lines denoting coupling strengths relative to the reorganization energy of the pentacene molecule. Blue lines depict couplings of $\frac{\lambda }{100}\leq H_{ab}<\frac{\lambda }{10}$, green lines depict couplings of $\frac{\lambda }{10}\leq H_{ab}<\frac{\lambda }{2}$, and red lines depict couplings of $\frac{\lambda }{2}\leq H_{ab}$. The bottom row, panels K−O, depicts an isosurface of the hole carrier wavefunction, Ψ(*t*) (Equation 2), during FOB‐SH simulation of charge transport (red and blue). The crystallinity is an indication of the structural order of the system and was calculated from linearly interpolating between the density of amorphous and single‐crystalline phases.

Samples of bulk pentacene with various degrees of crystallinity were created through the melting of a block of 3000 pentacene molecules followed by subsequent quenching to room temperature for quench times of 0 ns (instant quench), 1, 10, and 100 ns, see Section 4 for simulation details. The resultant nanoscale morphologies of the samples are shown in **Figure** **1**A–E, where we have also included the structure of single‐crystalline pentacene, corresponding to the limit of an infinitely long quench time. The 0 ns quenched system is fully amorphous (Figure 1A), as indicated by the flat angular (Figure S1, Supporting Information) and radial distributions (Figure S2, Supporting Information) between pentacene molecules. The density of the amorphous sample (ρ_am_ = 1.19 g cm^−3^) is 91% of the density of single‐crystalline pentacene (ρ_cr_ = 1.30 g cm^−3^), which is close to typical values reported in experiment, 87 %.^[^ ^22^ ^]^ At fast quench times of 1 ns, we observe simultaneous seeding in many regions of the sample leading to the formation of small ordered structures (Figure 1B). As each of the seeds became larger they blocked the path of neighboring fragments and prevented the growth of any larger ordered structures. This gave rise to the formation of many crooked and short 1D channels of ordered pentacene (approximately five molecules), which are randomly oriented with respect to one another. At this point, the crystallinity of the sample defined in terms of the mass density, ρ, is $Cr\;=\;(\rho -\rho_{\text{am}})\text{/}(\rho_{\text{cr}}-\rho_{\text{am}})\cdot 100\;\;\;=\;\;30\%$.

As the quench time increases so too does the tendency to form well ordered layers within the sample. After 10 ns quench time, we observe the formation of ordered pentacene layers stacked head‐to‐toe resulting in small grains of dimension 5–10 nm that are randomly oriented with respect to one another (Figure 1C, Cr = 60%). Importantly, we see that each layer forms a 2D herringbone pattern, the hallmark of the structure of single‐crystalline pentacene, with a characteristic peak in the angular distribution function at θ = 52.0°, close to the experimental value for the single crystal, θ = 54.3° (Figure S1, Supporting Information). In some layers, we observe crystal growth in two different directions, separated by a grain boundary, as clearly seen in Figure 1M. Finally, at 100 ns quench time, virtually all herringbone layers are stacked head‐to‐toe forming an ordered 3D structure that is already very similar to the one for single‐crystalline pentacene (Figure 1D, Cr = 80%). However, several imperfections are still clearly visible, in particular in the centre of the sample where two herringbone layers intersect two other layers.

#### 2.1.1 Electronic Coupling Maps

Turning to electronic properties, the first question that comes to mind is how the different nanoscale morphologies impact the electronic coupling between the pentacene molecules. Here we analyze representative 2D cuts through the samples (Figure 1F–J), corresponding to the areas coloured in gray in Figure 1A–E. The centres of mass of each molecule are joined with lines according to the strength of electronic coupling (*H*_*ab*_) between them. Blue, green and red lines depict small, medium and high coupling strengths, λ/100 ≤ *H*_*ab*_ < λ/10, λ/10 ≤ *H*_*ab*_ < λ/2 and λ/2 ≤ *H*_*ab*_, respectively, where λ is the molecular (or inner‐sphere) reorganization free energy of pentacene, 98 meV. If the picture of hole hopping between molecules was applicable, the blue and green lines would correspond to ET steps in the non‐adiabatic and adiabatic regime, respectively. For all red connections standard ET theory breaks down because at this point electronic coupling is so strong that there is no longer an activation barrier between (energetically degenerate) initial and final states and the charge carrier fully delocalizes over both sites.^[^ ^23^ ^]^ Extended hopping theories accounting for charge carrier delocalization could provide a remedy for this situation.^[^ ^19^ ^]^ As expected, we observe that the sample becomes electronically better connected (more red connections) as the crystallinity increases. We quantify this by clustering regions of high couplings as sets of *N* molecules that can all be connected with an uninterrupted path of red lines (see **Table** **1** for a summary). In the amorphous sample (0 ns) we observe formation of small islands of size 4 ± 4 molecules. At 30% crystallinity these islands become connected resulting in the formation of elongated 1D paths, which extend to 2D clusters at 60% crystallinity. At 80% crystallinity these clusters grow to 9 ± 16 molecules, but still short of the formally infinitely large cluster size of the single crystal. The notably wide spread in cluster size distribution is due to the presence of a large number of smaller clusters (two to four molecules). As we will discuss further below, they have a marked impact on electron hole delocalization and mobilities.

**Table 1:** Properties of pentacene in different bulk structures and in ultrathin (2D) films

Bulk pentacene							
τ (ns)^a)^	Structure^b)^	ρ (g cm^−3^)^c)^	*Cr* (%)^d)^	*N* ^e)^	IPR^f)^	μ^g)^, ^h)^ (comp)	μ^h)^ (exp)
0	am	1.19	0	4 ± 4	3.0	0.21	
1	nc	1.22	30	5 ± 5	3.8	0.23	
10	nc	1.25	60	7 ± 9	4.8	0.92	
100	nc	1.28	80	9 ± 16	9.5	1.8	
(∞)	sc	1.30	100	∞	17	10	5^i)^; 5.6^j)^
2D pentacene							
	sc, 1L			∞	5.4	4.2	1.6^k)^
	sc, 2L			∞	12	7.3	3^k)^

^a)^ Quench time from 800 to 300 K in molecular dynamics simulation in the NPT ensemble

^b)^ am, amorphous; nc, nanocrystalline; sc, single crystalline; 1L, 1 wet layer + 1 sc monolayer; 2L, 1 wet layer + sc bilayer

^c)^ Mass density

^d)^ Crystallinity, see main text for definition

^e)^ Mean and root‐mean‐square fluctuation of number of molecules in clusters with high coupling, see main text

^f)^ Equation (6), from FOB‐SH simulation

^g)^ Largest eigenvalue of charge mobility tensor obtained from FOB‐SH simulation, $\mu \;=\;\max(\mu_{ii}^{diag})$, *i* = 1,2,3 (Equation 4); The elements of the diffusion tensor are obtained from a linear fit of *MSD*_*αβ*_ (Equation 5) between typically 200–300 fs and about 1 ps. For disordered samples the mobilities reported were averaged over different regions of the sample

^h)^ In units of cm^2^ V^−1^ s^−1^

^i)^ Ref. [45], OFET, single crystal on Al_2_O_3_+ionic liquid, polymorph I

^j)^ Ref. [16], OFET, thin single crystal on SiO_2_

^k)^ Ref. [24], OFET, ultrathin (2D) single crystal on boronitride.

#### 2.1.2 Quantum (De)localization of Charge Carrier

We have carried out FOB‐SH non‐adiabatic molecular dynamics simulation of electron hole transport for the amorphous, nanocrystalline, and single‐crystalline pentacene samples at room temperature. The theoretical background and the computational details for these simulations are given in Section 4. We first consider representative snapshots of the carrier wavefunctions along FOB‐SH trajectories, as illustrated in Figure 1K–O. It is clearly visible that the delocalization of the wavefunction, defined in terms of the inverse participation ratio (IPR; Equation 6), increases with increasing crystallinity, reflecting the trend seen in the electronic coupling maps. In the amorphous sample, the static disorder of electronic couplings results in the wavefunction localizing, on average, over just two to three molecules. At 30% and 60% crystallinity, the high concentration of defects restricts wavefunction delocalization over 5–6 molecules, whereas at 80% we observe a marked increase to 10 molecules, which is still some way off from the value for the single crystal, 17 molecules. At 80% crystallinity we observe for the first time a clear spatial anisotropy of the charge carrier wavefunction extending more strongly along the T1 high coupling direction in the pentacene crystal (along the diagonal in Figure 1N). For samples up to 80% crystallinity we also notice a remarkably good correlation between the IPR and the cluster size *N* in the electronic coupling maps (Table 1), suggesting that carrier delocalization is limited by the static disorder of electronic coupling. This correlation is lost for the single crystal because in this case charge carrier delocalization is limited by the dynamic (or thermal) disorder of electronic couplings.

### 2.2 Charge Transport Mechanism

**Figure 2:**
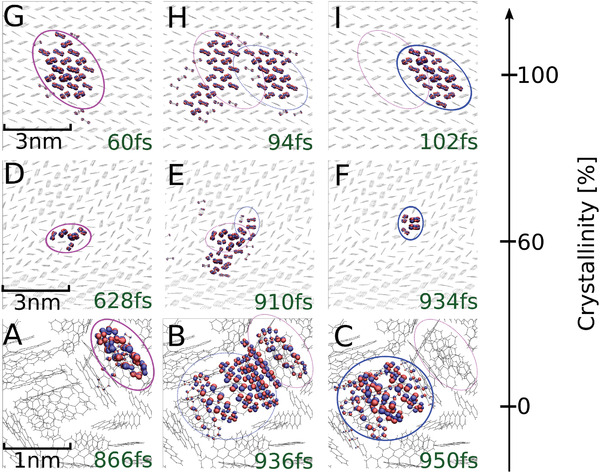
Mechanism of hole transport in bulk pentacene phases. The transport scenario for amorphous, nanocrystalline and single‐crystalline pentacene are shown in panels A−C, D−F and G−I, respectively. Pentacene molecules are shown in grey stick representation and the crystallinity of the phases is indicated on the scale to the right. Isosurfaces of the charge carrier wavefuntion, Ψ(*t*) (Equation (2)), are depicted in red and blue colors for three different times along a FOB‐SH trajectory as indicated. The initial positions and extensions of the hole polaron are shown in the snapshots to the left (circles in pink), the transitions to the new positions are shown in the snapshots in the middle, and the hole polaron in the new position is shown in the snapshots to the right (circles in blue).Notice the different extent of hole carrier delocalization for the different phases. See main text for a detailed description of the mechanisms.

Analyzing the FOB‐SH trajectories, we observe three qualitatively different charge transport mechanisms depending on the crystallinity of the sample. In the amorphous sample (**Figure** **2**A–C), the polaron is relatively localized (albeit not fully localized on a single site) and is observed to hop infrequently from one small island to the next via transient delocalization over and relocalization on the new island, reminiscent of the charge hopping mechanism that is often assumed for disordered structures. There is no preferential direction for hopping, the transport is slow and isotropic. The situation is markedly different at 60% crystallinity (Figure 2D–F) In this system, as a consequence of multiple crystal domains forming, the transport mechanism depends strongly on the initial position of the charge carrier wavefunction. If initialized within a region of high static disorder (e.g., within a grain boundary), the polaron is typically delocalized over just a few molecules (Figure 2D). Under the influence of thermal nuclear motion, the polaron temporarily expands to neighboring molecules in the crystalline domains (Figure 2E), but eventually collapses to a state in the grain boundary (Figure 2F). When initialized within a crystalline domain, the polaron is initially strongly delocalized, similarly as in the single crystal, but eventually gets trapped in a region of high static disorder. The relatively localized electronic states in these disordered regions are located close to the top of the valence band and thus act as polaron traps that make the transport sluggish. Our FOB‐SH simulations correctly describe this effect because they obey, to a very good approximation, detailed balance, that is, Boltzmann sampling of the electron hole states in the valence band in the limit of long simulation times. Finally, in the single crystal, the delocalized charge carrier frequently expands to more than twice its original size, preferably along the high coupling direction T1 within the herringbone layer, followed by collapse to its original size at a neighboring region in the crystal (Figure 2G–I). These “diffusive jumps” of a “flickering” polaron as we previously called them^[^ ^13^ ^]^ displace the centre of charge of the polaron by several lattice spacings at a time resulting in high (and anisotropic) charge mobilities.

#### 2.2.1 2D Pentacene

In addition to bulk samples we have also investigated ultrathin (2D) films, which have attracted considerable interest as a platform for new device structures.^[^ ^24^ ^]^ In a recent work, the fabrication of single‐crystalline 2D pentacene films was reported that consisted of only four layers,^[^ ^24^ ^]^ as illustrated in Figure S3, Supporting Information: a boronitride substrate; a wetting layer of pentacene molecules laid parallel to the substrate; and two highly ordered layers of pentacene molecules stacked such that their long axis forms an angle of 61° (1L) and 82° (2L) with respect to the plane of the substrate. We have modeled these ultrathin film structures and carried out FOB‐SH non‐adiabatic MD simulation to understand how the charge transport mechanism compares to our results for the bulk samples described above. We observe that the charge carrier, once initialized in a given layer (1L or 2L), remains in that layer and does not cross over to the other layers as the electronic coupling between them is very small (0.5 meV), similar to the situation in bulk. While the charge carrier delocalization is somewhat smaller than in bulk single‐crystal pentacene due to different packing and somewhat smaller electronic couplings (5.4 molecules for 1L and 12.0 molecules for 2L), the transport mechanism within the layers is very similar to the flickering polaron scenario described above for bulk single crystals.

### 2.3 Electron Hole Mobilities

**Figure 3:**
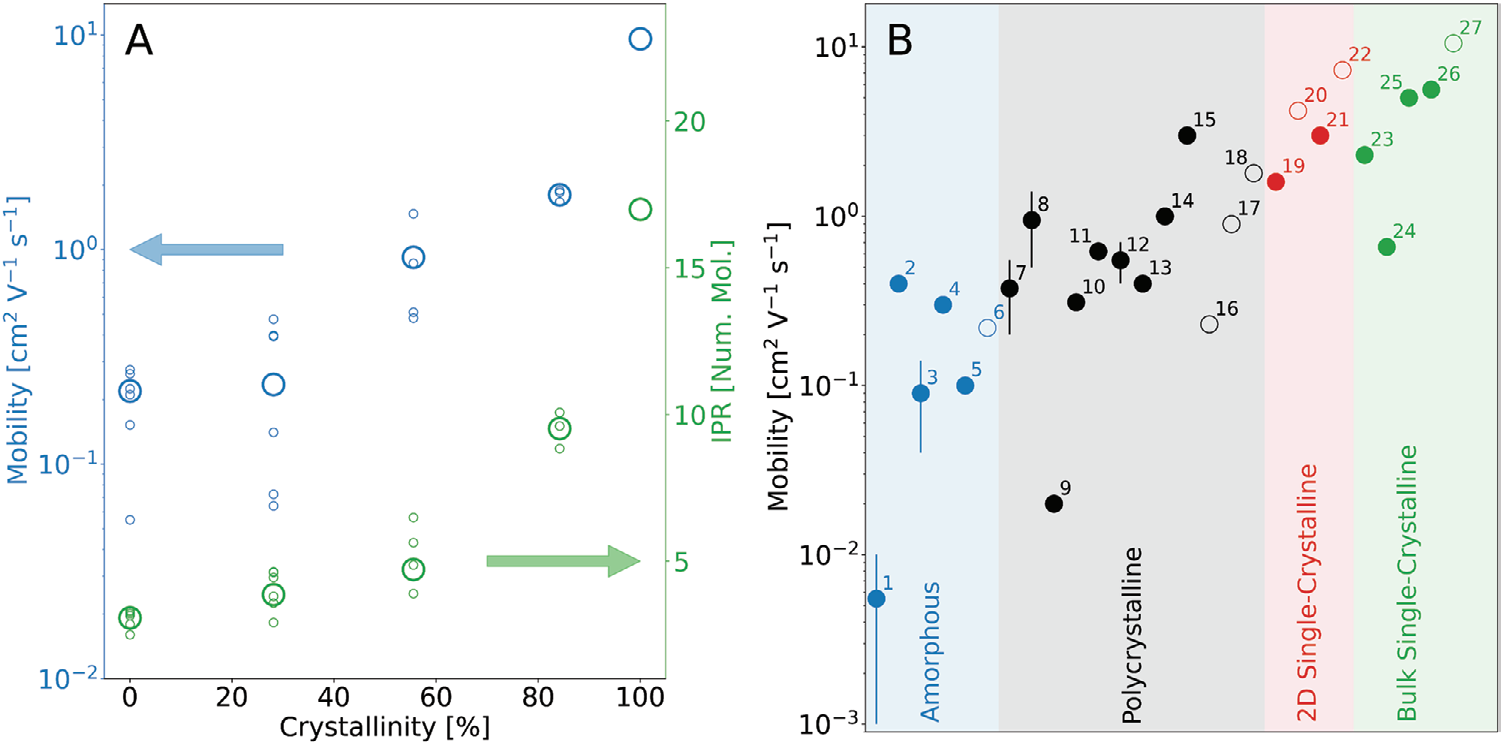
Hole mobilities and inverse participation ratio (IPR) for the pentacene phases studied. A) Hole mobilities and IPR from FOB‐SH simulation are shown for bulk pentacene phases as a function of the crystallinity of the sample (blue and green circles, respectively). The local mobilities and IPR for different regions of the sample are shown in small circles and the averages are shown in large circles. B) FOB‐SH mobilities for bulk pentacene phases and 2D pentacene layers (open symbols) are compared to experimental results (filled symbols). The bulk pentacene phases are classified as “amorphous”, “polycrystalline,” and single crystalline. Error bars for computed values indicate the spread in local mobility. 1,2: ref. [48], 3: ref. [49], 4,5: ref. [50], 6: this work, *Cr* = 0%, 7,8: ref. [51], 9,10,11: ref. [52], 12: ref. [53], 13,14,15: ref. [54], 16: this work, *Cr* = 30%, 17: this work, *Cr* = 60%, 18: this work, *Cr* = 80%, 19: ref. [24], 20: this work, 2D pentacene, 1L, 21: ref. [24], 22: this work, 2D pentacene, 2L, 23,24: ref. [55], 25: ref. [45], 26: ref. [16], 27: this work, *Cr* = 100%. Additional information on the device measurements and gate dielectrics used can be found in Table S1, Supporting Information.

Hole mobilities for all pentacene samples discussed above were obtained from the mean‐square displacement of the charge carrier wavefunction as a function of time, averaged over a few hundred FOB‐SH trajectories (Figure S4, Supporting Information). For the disordered systems we divided the full sample of 3000 molecules in up to six regions of equal size (Figures S5 and S6, Supporting Information) and calculated the charge mobility for each of them separately. These “local” charge mobilities inform us of the impact of structural inhomogeneity of the quenched samples on charge transport. We find that in the disordered samples, especially the one with ≈30% crystallinity, the local charge mobilities and IPR values exhibit a relatively large spread as some regions are more crystalline and thus more conductive than others (**Figure** **3**A, small open circles). In the structurally more homogenous sample with 80% crystallinity, the variation in local mobility becomes almost negligible. The average of the local charge mobilities and IPRs correlate well with the crystallinity of the sample (Figure 3A, large circles).

Over the last 20 years a large number of experimental hole mobilities have been reported for pentacene thin films and crystals from OFET measurements. Yet, there are several issues to consider when comparing our calculations to these measurements. In OFETs charge transport is typically probed on the micrometer scale over macroscropic time scales, whereas present FOB‐SH simulations are carried out for nanoscale samples over nanoseconds of accumulated simulation time. Moreover, OFET mobilities have been shown to be very sensitive to many details of the preparation method including, for example, the gate dielectric used, the surface roughness, deposition rate, and temperature. For comparison with present computations, we grouped the experimental measurements in four categories depending on the structural morphology of pentacene: amorphous, polycrystalline, 2D single crystalline, and bulk single crystalline, see Figure 3B and Table S1, Supporting Information for numerical values and references. Recent measurements for amorphous samples gave 0.04–0.3 cm^2^ V^−1^ s^−1^ depending on the deposition rate, compared with an average of 0.2 cm^2^ V^−1^ s^−1^ from our FOB‐SH simulations. OFET mobilities for polycrystalline samples typically range between 0.2 and 1.4 cm^2^ V^−1^ s^−1^, which compares well with our computed range of average values, 0.2–1.8 cm^2^ V^−1^ s^−1^, for nanocrystalline samples of 30–80% crystallinity. The reported mobilities for 2D and bulk single‐crystalline pentacene are 1.6–3 and 2.3–5.6 cm^2^ V^−1^ s^−1^ compared to 4.2–7.3 and 9.6 cm^2^ V^−1^ s^−1^ from present calculations. Hence, notwithstanding the above caveats, the correlation between experiment and computed FOB‐SH mobilities is rather good, which supports the mechanistic picture that our simulations have revealed.

## 3 Conclusion

We have shown that it is now possible to use explicit quantum dynamical calculations to simulate charge carrier transport in large, realistic samples of disordered organic semiconductors. Our results are in remarkably good agreement with those available from experiment and provide a molecular‐scale picture of the nature of the charge carrier and the transport mechanism as a function of the crystallinity of the system. The notion that charge carrier transport in disordered systems occurs via hopping of relatively localized polarons is shown to be a reasonably good approximation only for perfectly amorphous systems—for nanocrystalline samples significant charge carrier delocalization occurs mandating the use of more advanced transport simulations, for example, the FOB‐SH method used here. In general, there is a good correlation between crystallinity, carrier delocalization, and mobility. Interestingly, we find that even relatively small amounts of structural disorder can lead to a significant drop in charge carrier delocalization and hole mobility compared to the single crystal. This is an important consideration when comparing charge carrier mobilities in simulated organic systems, usually perfectly crystalline, with those of experiment, where it is difficult to prepare highly pure crystals devoid of defects. Our approach is generally applicable to any molecular organic semiconductor and may be used in future work for identifying new disordered materials with high charge mobility.

## 4 Experimental Section

### Preparation of Pentacene Structures

Disordered bulk samples of pentacene were generated with a melt‐quench procedure.Initially, 3000 pentacene molecules were placed on a regular 3D grid inside an orthorhombic unit cell and melted to a temperature of 800 K. Velocities were initially randomly sampled from a Gaussian distribution corresponding to this temperature and a Nosé–Hoover thermostat and a barostat (target pressure 1 bar) were used to control temperature and pressure in the isothermal–isobaric ensemble (NPT). After 1 ns, the temperature was linearly decreased to 300 K over quench times of 0, 1, 10, and 100 ns. Finally, a 1 ns NPT equilibration run was carried out at 300 K followed by a short 0.25 ns run in the NVT ensemble, applying the last cell dimensions from the preceding NPT run. Standard intramolecular and Van der Waals interactions of the general AMBER force field^[^ ^25^ ^]^ (GAFF) were used to model pentacene, which have been well used and justified in a number of studies.^[^ ^26^, ^27^, ^28^, ^29^, ^30^, ^31^, ^32^ ^]^ Electrostatic interactions were modeled by restrained electrostatic potential^[^ ^33^ ^]^ (RESP) partial charges obtained from B3LYP level of theory using a 6‐311g(d) basis set. The melt‐quench simulations were carried out with the LAMMPS molecular dynamics package^[^ ^34^, ^35^ ^]^ employing the particle‐particle‐particle‐mesh Ewald method for calculation of the electrostatic interactions.^[^ ^36^ ^]^ The B3LYP calculations were carried out with the Gaussian Programme.^[^ ^37^ ^]^ Single‐crystalline pentacene was created by repeating the triclinic unit cell taken from the Cambridge Structural Database (CSD)^[^ ^38^ ^]^ to form a 20 × 40 molecule plane and equilibrating to 300 K in the NVE ensemble, for 1 ns, using the experimental cell dimensions. The 2D ultrathin film structure was reconstructed from the model structure of Zhang et al.^[^ ^24^ ^]^ The latter was obtained from experimental data and corroborated by DFT optimizations, see Supporting Information for more details.

### FOB‐SH Non‐Adiabatic Molecular Dynamics of Hole Transport

The FOB‐SH methodology has been described in detail in a series of previous papers.^[^ ^13^, ^39^, ^40^, ^41^, ^42^ ^]^ Here only a very brief summary of the relevant equations was given. The valence band of the pentacene sample is described by the following Hamiltonian,

(1) $$\begin{array}{c} H=\underset{k}{\sum }\varepsilon_{k}|\phi_{k}\rangle \langle \phi_{k}|+\underset{k\neq l}{\sum }H_{kl}|\phi_{k}\rangle \langle \phi_{l}| \end{array}$$

where ϕ_*k*_ = ϕ_*k*_(**r**, **R**(*t*)) is the HOMO of molecule *k*, **r** is the position v of the ehole, **R**(*t*) are the time‐dependent nuclear coordinates, ε_*k*_ = ε_*k*_(**R**(*t*)) is the site energy, that is, the potential energy of the state with the hole located at site *k* and *H*_*kl*_ = *H*_*kl*_(**R**(*t*)) is the electronic coupling between ϕ_*k*_ and ϕ_*l*_. All Hamiltonian matrix elements, that is, site energies and couplings, depend on the nuclear coordinates which, in turn, depend on time, **R = R**(*t*) as determined by the nuclear dynamics. As shown before, the electronic Hamiltonian Equation (1) reproduces very well the DFT valence band structure of single‐crystalline pentacene.^[^ ^13^ ^]^ In the FOB‐SH approach the hole was described by a time‐dependent 1‐particle wavefunction, Ψ(*t*), expanded in the same basis that was used to represent the Hamiltonian Equation (1)

(2) $$\begin{array}{c} \psi (t)=\sum_{l=1}^{M}u_{l}(t)\phi_{l}(R(t)) \end{array}$$

where *u*_*l*_ are the expansion coefficients. Insertion of Equation (2) in the time‐dependent Schrödinger equation gives the time evolution of the hole carrier wavefunction in the valence band

(3) $$\begin{array}{c} i\hbar {\dot{u}}_{k}(t)=\sum_{l=1}^{M}u_{l}(t)\left(H_{kl}(R(t))-i\hbar d_{kl}(R(t))\right) \end{array}$$

where $d_{kl}\;\;=\;\langle \;\phi_{k}|{\dot{\phi }}_{l}\rangle$ are the non‐adiabatic coupling elements. The nuclear degrees of freedom were propagated on one of the potential energy surfaces (PES) obtained by diagonalizing the Hamiltonian Equation (1) and denoted as *E*_*a*_ (“*a*” for “active surface”). While nuclear motion coupled to the motion of the hole via the dependences on **R**(*t*) in Equation (3), feedback from the hole to the nuclear motion was accounted for by transitions of the nuclear dynamics (“hops”) from the PES of the active eigenstate *a* to the PES of another eigenstate *j* using Tully's surface hopping probability.^[^ ^43^ ^]^

### FOB‐SH Simulation Details

FOB‐SH simulation of hole transport was carried out for different regions of the quenched structures. For the amorphous structures obtained after 0 and 1 ns quench time (*Cr* = 0 and 30%, respectively) FOB‐SH simulations were carried out for six rectangular regions as indicated in Figure S5, Supporting Information. A thin wrapper of nearest neighbor molecules was also included to improve energy conservation and maintain the subsystem's structure. For the nanocrystalline structures obtained after 10 and 100 ns quench time (*Cr* = 60 and 80%, respectively) FOB‐SH simulations were carried out for four, respectively, three crystal planes, isolated via a density‐based clustering algorithm, as indicated in Figure S6, Supporting Information. For a given region, 500–750 molecules were chosen to be treated electronically active, that is, they were treated as molecular sites for construction of the electronic Hamiltonian Equation  (1), with their HOMO contributing to the expansion of the carrier wavefunction Equation  (2). All other molecules in the 3000 molecule supercell were treated electronically inactive and interacted with the active region only via non‐bonded interactions. From the equilibrated trajectory, an uncorrelated set of nuclear positions and velocities were chosen as starting configurations for FOB‐SH simulations. The initial hole carrier wavefunction was chosen to be an eigenstate of the Hamiltonian Equation  (1), located at the centre of the electronically active region and within about 2*k*_B_ *T* from the top of the valence band. The hole carrier wavefunction and nuclei were propagated in time according to the FOB‐SH algorithm (see above) in the NVE ensemble. The simulations were carried out as described previously for single‐crystalline pentacene^[^ ^13^ ^]^ using decoherence correction,^[^ ^42^ ^]^ removal of decoherence‐induced spurious long‐range charge transfer,^[^ ^41^, ^42^ ^]^ adjustment of the velocities in the direction of the non‐adiabatic coupling vector in case of a successful surface hop,^[^ ^40^ ^]^ trivial crossing detection^[^ ^41^, ^42^ ^]^ and the multiple time step algorithm.^[^ ^13^ ^]^ The nuclear time step was 0.05 fs. The electronic time step for integration of Equation  (3) using a fourth‐order Runge–Kutta algorithm was 0.01 fs. For each region between 220 and 500 FOB‐SH trajectories of length 0.5–1.5 ps were run to obtain the time‐dependent mean‐square displacement of the charge carrier wavefunction, the diffusion tensor (Equation 5) and the charge mobility tensor (Equation 4), see below for details. Similar calculations were carried out for the single‐crystalline and 2D single‐crystalline structures, with active regions chosen large enough to converge charge mobility, 800 molecules for bulk single crystal, and 782 and 900 molecules for 2D single‐crystal 1L and 2L, respectively. In the bulk single‐crystalline system the charge was initialized in a bottom corner of the active region to allow the mobility to converge before encountering an edge and to travel along the T1 (high mobility) direction. All simulations were carried out with our in‐house implementation of FOB‐SH in the CP2K simulation package.^[^ ^44^ ^]^

The charge mobility μ of a disordered region of a quenched structure or of a single‐crystalline sample was taken to be equal to the largest eigenvalue of the mobility tensor, $\mu \;=\;\max(\mu_{ii}^{diag})$, *i* = 1,2,3. The latter was calculated from the Einstein relation

(4) $$\begin{array}{c} \mu_{\alpha \beta }=\frac{eD_{\alpha \beta }}{k_{\text{B}}T} \end{array}$$

where *D*_*αβ*_ is the diffusion tensor

(5) $$\begin{array}{c} D_{\alpha \beta }=\frac{1}{2}\underset{t\to \infty }{\lim}\frac{d{\text{MSD}}_{\alpha \beta }(t)}{dt} \end{array}$$

and MSD_*αβ*_ is the mean‐square displacement of the charge carrier wavefunction Ψ(*t*) obtained from FOB‐SH simulation (see ref. [13] for an explicit expression). After initial relaxation, typically 200–300 fs, the components of the MSD increase linearly in time, to a good approximation. Linear fits for all components MSD_*αβ*_ were made according to Equation (5). The *R* ^2^ values of the fits for the two largest components of the MSD tensor were in the range 0.72–0.99, and in the majority of cases >0.9; see, for example, Figure S4, Supporting Information. The convergence of the MSD with respect to the number of FOB‐SH trajectories was investigated by dividing the full set of trajectories into two subsets and calculating the charge mobility for each subset separately. Half of the deviation of the two mobility values relative to the mobility value obtained for the full set of trajectories, Δμ/(2μ) × 100 was reasonably small, between 2% and 33%, with the majority of cases <15% indicating that the number of trajectories run was sufficient.

The IPR was defined by

(6) $$\begin{array}{c} \text{IPR}=\frac{1}{T}\overset{T}{\underset{0}{\int }}dt\frac{1}{N_{trj}}\sum_{n=1}^{N_{trj}}\frac{1}{\sum_{k=1}^{M}|u_{k,n}{|}^{4}(t)} \end{array}$$

where *u*_*k*,*n*_ is the expansion coefficient *k* of the wavefunction Ψ(*t*) defined in Equation (2), *n* is the index of the trajectory, *M* the number of electronically active molecules in the sample, *N*_*traj*_ the number of trajectories, and *T* the length of the trajectories. The numerical value of the IPR is about equal to the number of the molecules the charge carrier wavefunction was delocalized over, averaged over time.

## Conflict of Interest

The authors declare no conflict of interest.

## Supporting information

Supporting Information

## Data Availability

The data that support the findings of this study are available from the corresponding author upon reasonable request. The data totals more than 1 TB, so is in cold storage accessible by the corresponding author.
