# Supplementary material for: Impact of Nanoscale Morphology on Charge Carrier Delocalization and Mobility in an Organic Semiconductor
Source: Adv Mater. 2021 Sep 23;33(45):2104852. doi: 10.1002/adma.202104852 (PMC11469049; doi:10.1002/adma.202104852)
Supplement: Supplementary file 1 — Supporting Information [file ADMA-33-2104852-s001.pdf]

© 2021 Wiley-VCH GmbH

ADVANCED MATERIALS

Supporting Information

for *Adv. Mater.,* DOI: 10.1002/adma.202104852

Impact of Nanoscale Morphology on Charge Carrier Delocalization and Mobility in an Organic Semiconductor

Matthew Ellis, Hui Yang, Samuele Giannini, Orestis G. Ziogos, and Jochen Blumberger**

Supplementary Information

1 Angular distributions of quenched pentacene structures

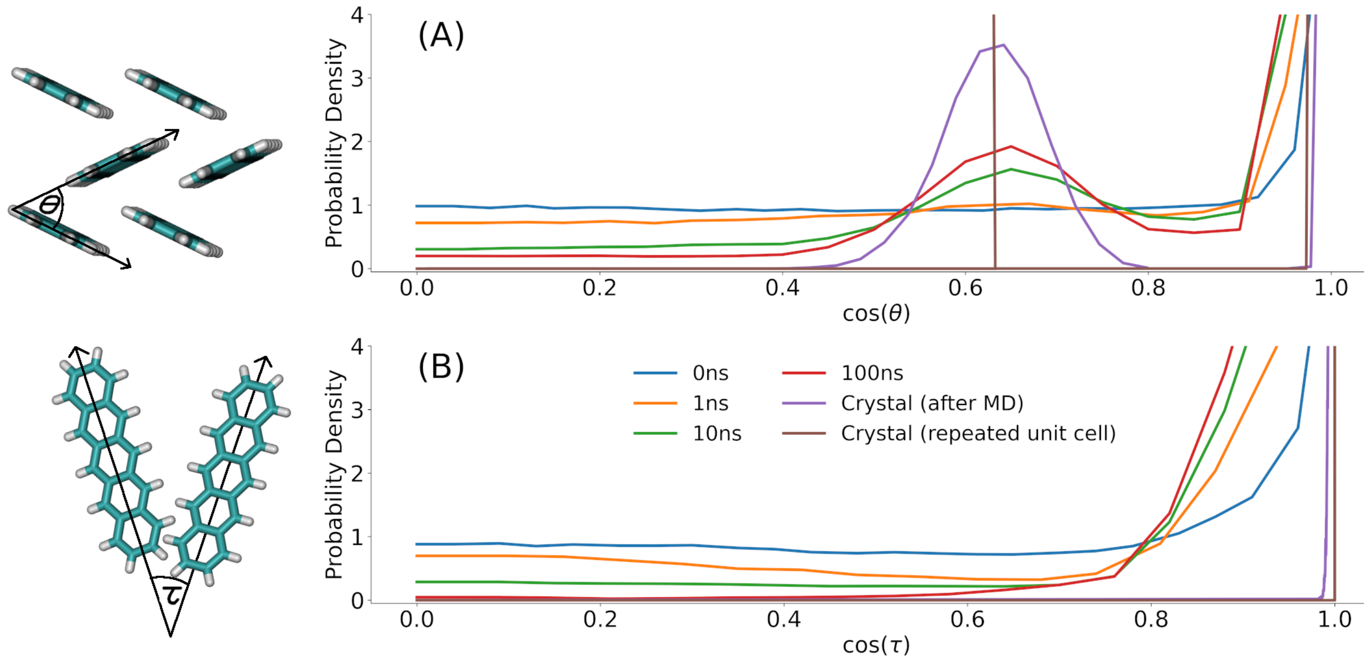

Figure S1: Distribution of short axis angle (θ, panel A) and long axis angle (τ, panel B). Experimental crystal structure of pentacene (brown),[1] after MD run of the experimental crystal structure at 300 K (purple), and for the 4 different melt-quench MD simulations of pentacene with quenching times as indicated (blue, orange, green, red). The angles are defined in the insets to the left.

2 Radial distribution functions of quenched pentacene structures

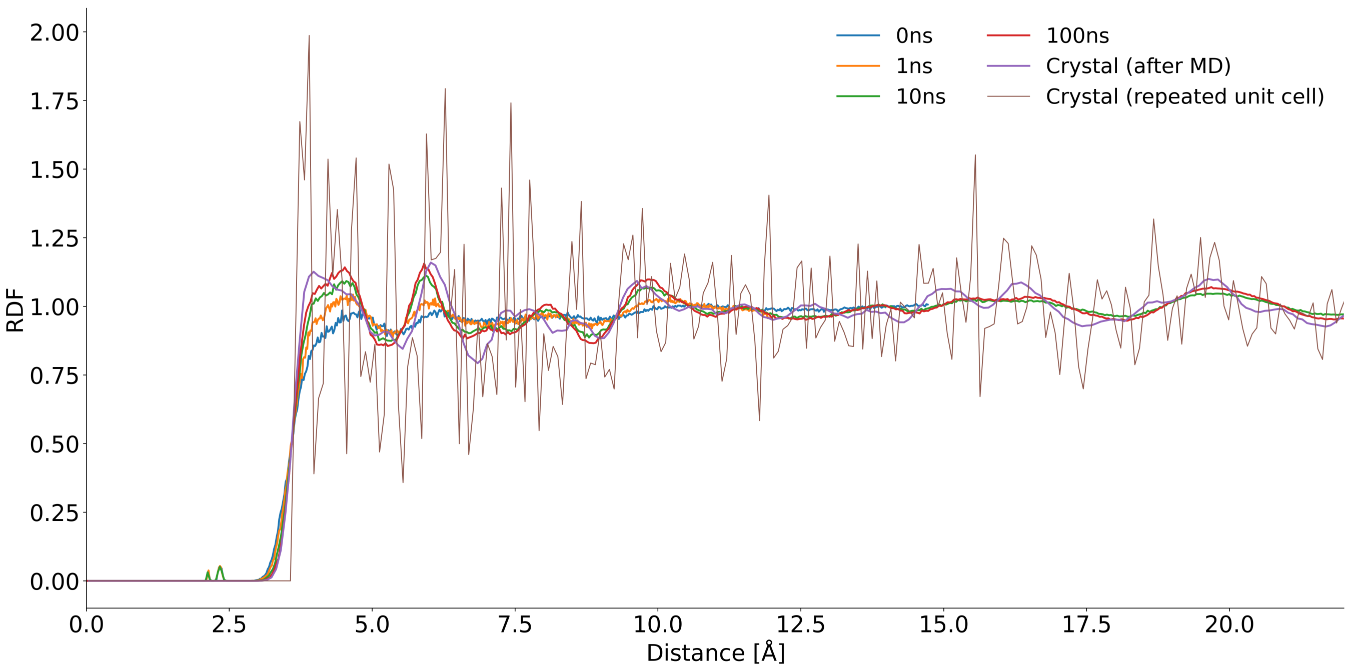

Figure S2: Radial distribution function of all pairs of carbon atoms on different molecules of pentacene. Experimental crystal structure of pentacene (shaded brown),[1] after MD run of the experimental crystal structure at 300 K (purple), and for the 4 different melt-quench MD simulations of pentacene with quenching times as indicated (blue, orange, green, red).

3 Thin film structure of pentacene

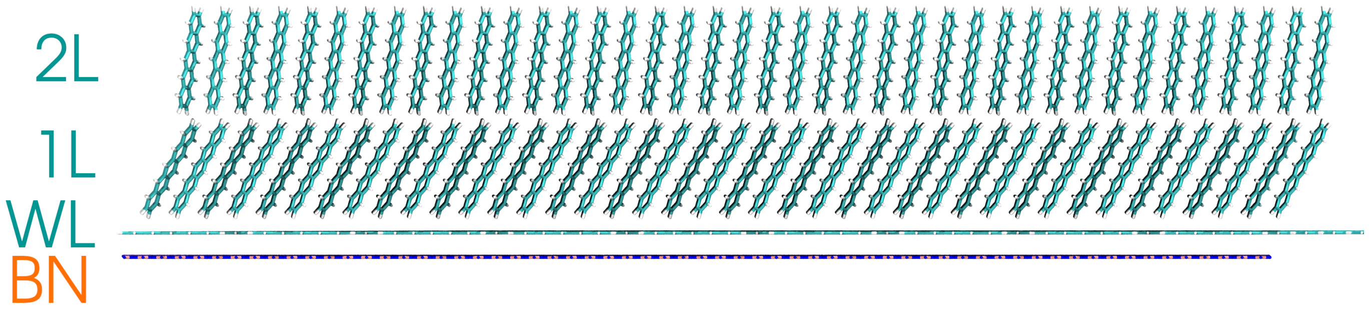

Figure S3: Model structure of 2D single-crystalline pentacene thin film.

The structure was reconstructed from the information provided in Ref.[2] which is based on experiment and corroborated by ab-initio DFT optimizations. Angles for 1L and 2L with respect to the principal axes in the Cartesian reference framework where taken from the SI of.[2] The pentacene wet layer (WL) was reconstructed considering that the pentacene molecules forming this layer are in a lower energy configuration when the hollow site of the pentacene rings coincide with B or N forming the boronitride (BN) layer. To study charge transport in this layered structure an extended and periodic supercell was created by replicating the unit cells of BN, WL, 1L and 2L. The 1L and 2L cells have no lattice mismatch as they share the same lattice parameters. The mismatch between BN, WL and 1L/2L was minimized as much as possible by replicating the 3 unit cells 20, 15 and 1 times resulting in dimensions along the a and b crystallographic directions of 155 Å and 90.93 Å, respectively. This supercell was then repeated once in the y direction to give the full structure. The total number of pentacene molecules in WL+1L+2L is 1326 plus two sheets of BN with 5208 atoms each. During MD and FOB-SH simulations we have used restraint potentials to preserve the tilt angle of the molecules within the 1L and 2L layers and to prevent any center of mass motion of each molecule. To this end, two centre of mass restraints were applied, one defined by the atoms of the first two benzene rings and one defined by the atoms of the last two benzene rings of pentacene. The restraint force constant was chosen as small as possible so as to provide the best compromise between maintaining structure and and keeping any bias on mobilities due to the restraint force at a minimum. This was determined by repeat simulations with successively decreasing restraint strengths and looking at the angular distributions, IPR and mobility of the charge carrier as a function of restraint force constant. An optimal value of 11.2 kcal mol^{-1} Å^{2} was chosen, which resulted in a stable structure with average tilt angles very close to the experimental values and a negligible change in mobility when the restraint strength was increased.

4 Mean Squared Displacements and IPR from FOB-SH simulation of charge transport

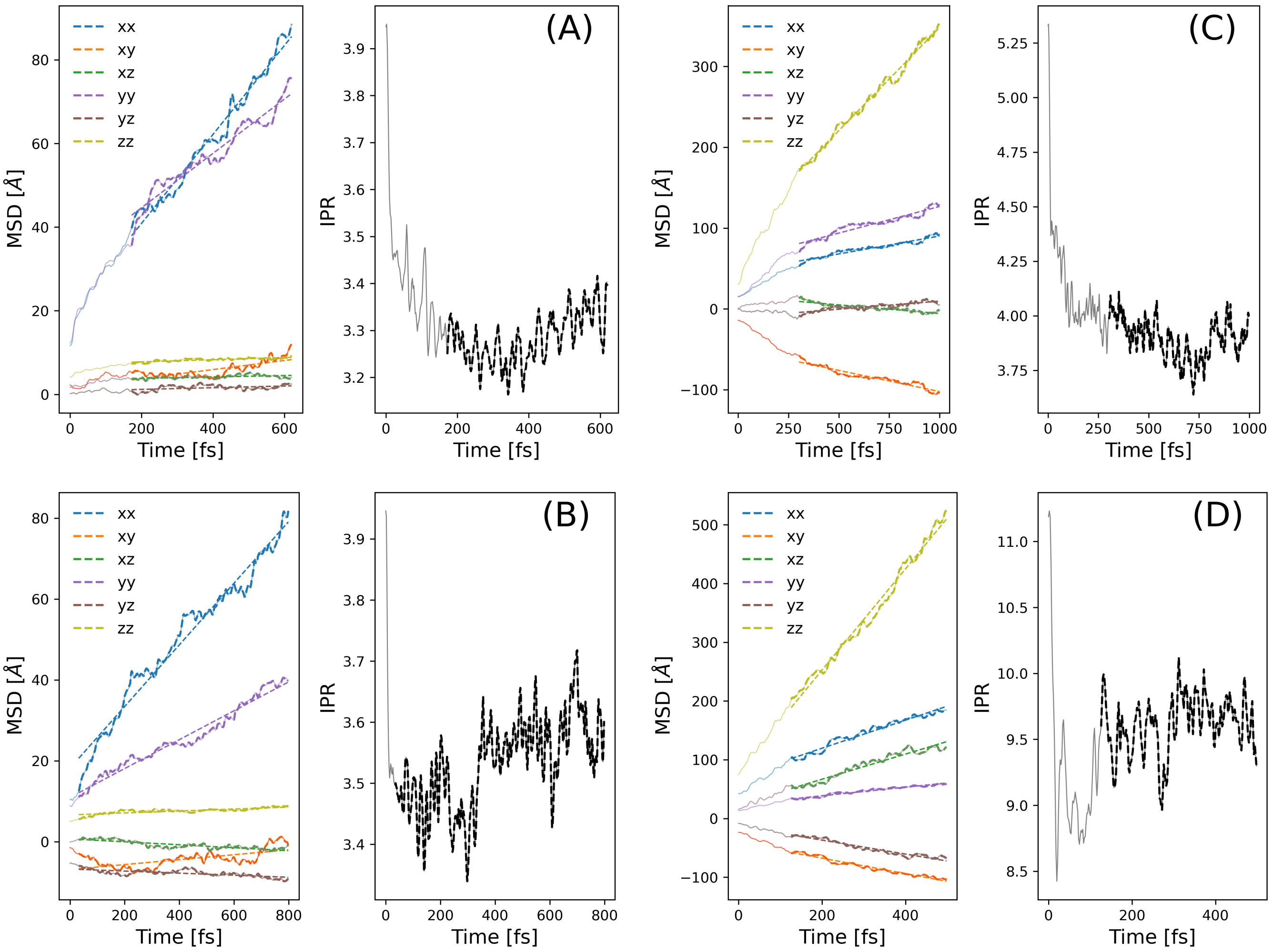

Figure S4: Representative MSD and IPR of the charge carrier in quenched pentacene samples from FOB-SH simulation. The quenching time of the samples was 0 ns (A), 1 ns (B), 10 ns (C) and 100 ns (D). The MSD tensor is calculated for each FOB-SH trajectory and averaged over trajectories. The 6 unique values within the MSD tensor are then plotted against time (left panel). After initial relaxation (thin lines), an approximately linear increase in the MSD is observed (thick lines). The corresponding diffusion tensor was obtained from linear fits of the MSD in the time intervals 170-620 fs (A), 30-798 fs (B), 300-1000 fs (C), 150-498 fs (D). The IPR was averaged similarly (right panel).

5 0 ns and 1ns quenched systems for FOB-SH simulation of charge transport

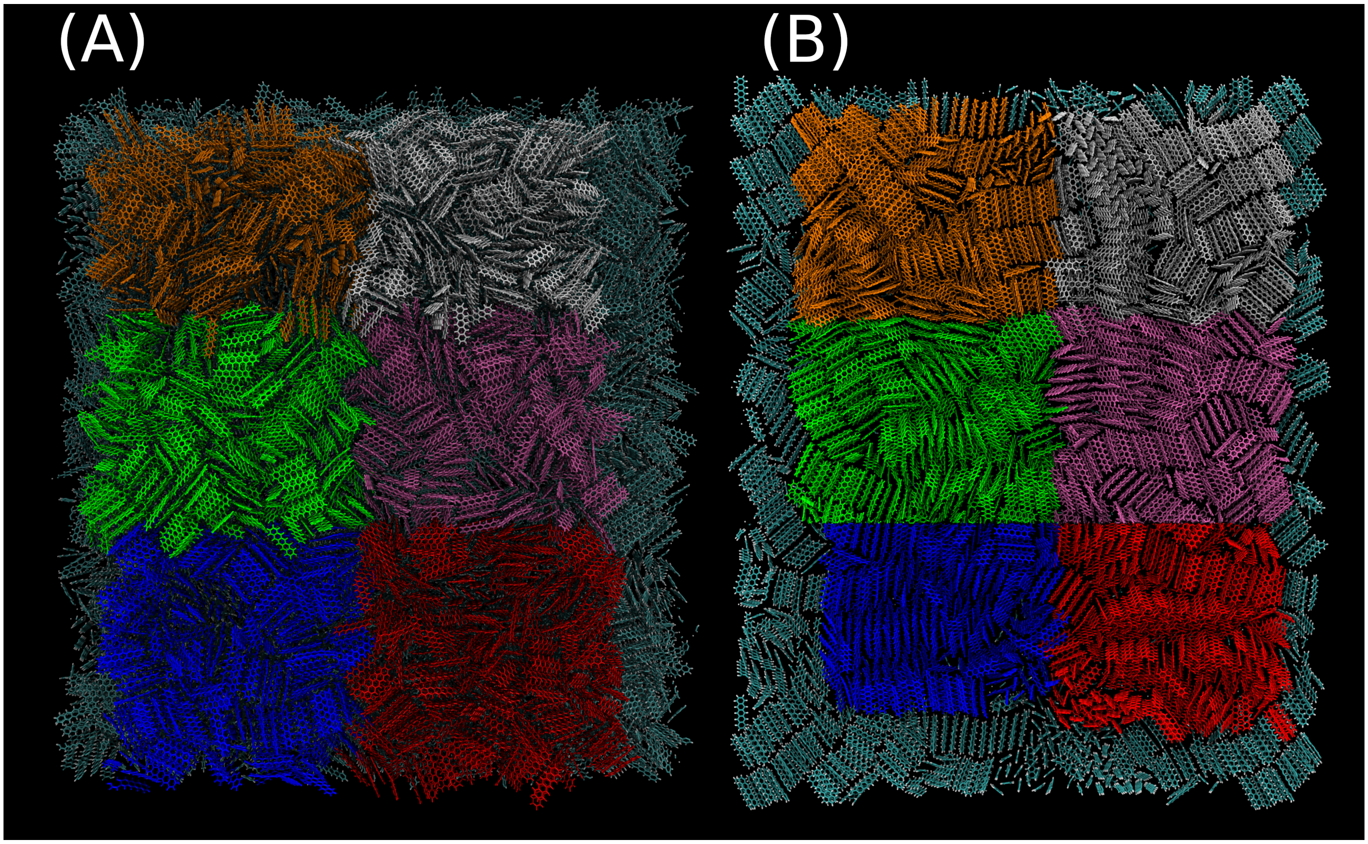

Figure S5: Six different regions for the 0 ns (panel A) and 1 ns (panel B) quenched structures for which FOB-SH simulation and local charge mobility simulations were carried out.

6 10 ns and 100 ns quenched structures for FOB-SH simulation of charge transport

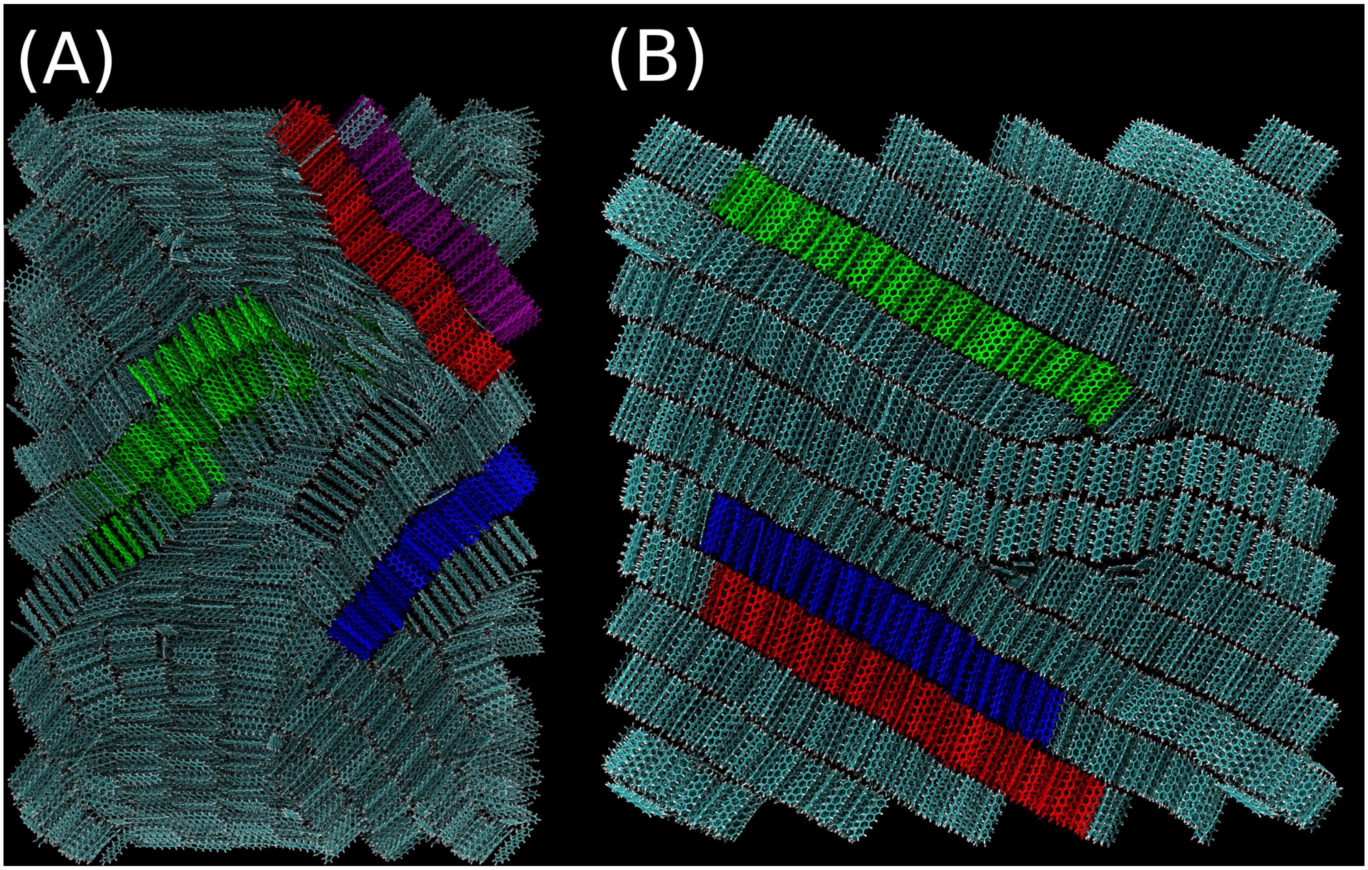

Figure S6: 4 different regions for the 10 ns (panel A) and 3 for the 100 ns (panel B) quenched structures for which FOB-SH simulation and local charge mobility simulations were carried out.

6.1 Orientational Order Parameter

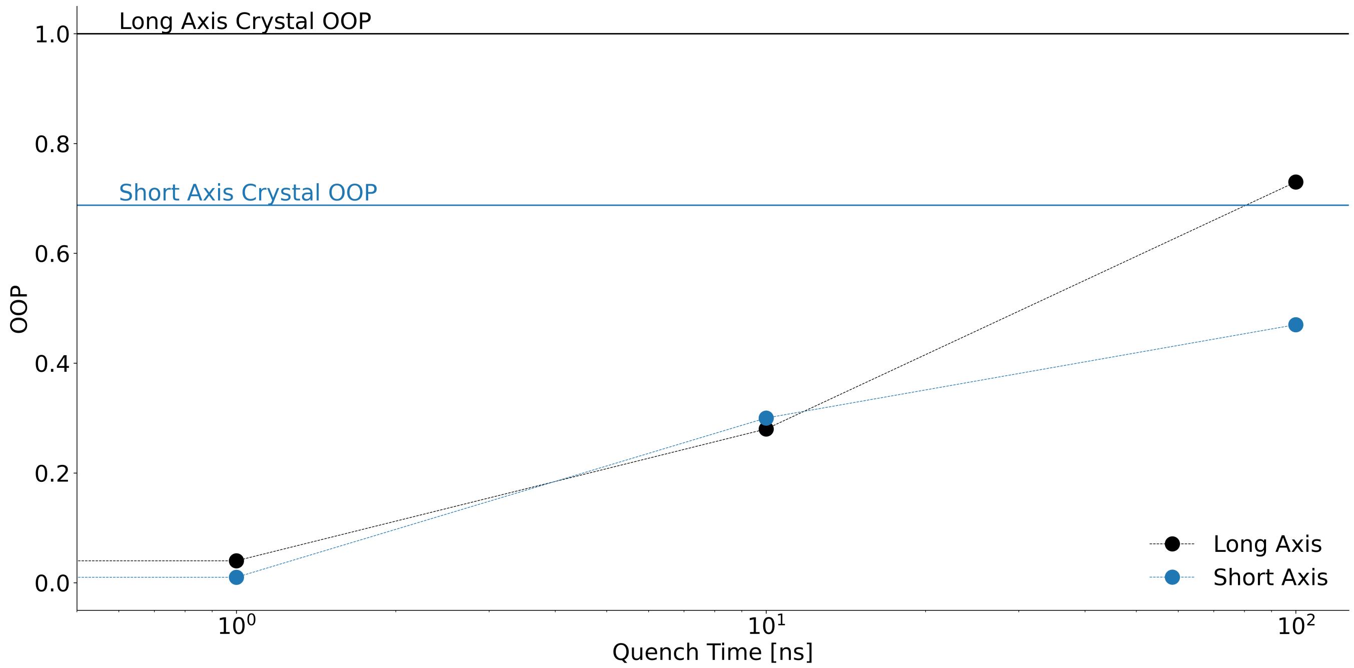

Figure S7: The change in the orientational order parameter (OOP) with respect to the quenching time. The black data represents the values for the long axis and the blue data represents the short axis values. The horizontal lines show the theoretical value for a perfect crystal.

The orientational order parameter gives a single number describing how aligned the molecules within a system are. Values lie on a scale from -0.5 to 1, where 1 denotes all molecules are aligned, 0 denotes a random alignment of molecules and -0.5 denotes an anti-alignment with respect to the reference vector. The formula for the orientational order parameter is given below in equation (1).

$$OOP = \frac{3}{2}\frac{1}{N_{mol}}\sum_{i}^{N_{mol}}\left[\frac{\mathbf{v}_i \cdot \mathbf{v}_{ref}}{|\mathbf{v}_i||\mathbf{v}_{ref}|}\right]^2 - \frac{1}{2} \tag{1}$$

Where $\mathbf{v}_i$ is the vector describing the long or short axis of molecule i. The reference vector $\mathbf{v}_{ref}$ was defined as the average over $\mathbf{v}_i$ i.e: $\mathbf{v}_{ref} = \langle \mathbf{v}_i \rangle_i$. N_{mol} is the number of molecules and i indicates a molecule index.

In Figure S7 we can see the change in the orientational order parameter with quenching time and, as seen in the previous sections, as we increase the quenching time we increase (orientational) order in the system. This is a quantification of the increase of order seen in the angular distribution in Figure S1.

Table S1: Experimental and computed charge mobilities for amorphous, polycrystalline and single-crystalline pentacene. References can be found in the main text.

Author	device/comp.	structure	gate dielectric	mobility	comments
1, Hesse80	Photocurrent	amorphous		0.001-0.01	long time mobility (1 μs)
2,				0.4	short time mobility (20 ns)
3, Bae13	TFT	amorphous	polymer	0.04-0.14	dependent on deposition rate
4, Choo02	TFT	amorphous	SiN_x/p-Si	0.3	
5		amorphous-crystalline		0.1	
6, this work	comp.	amorphous		0.2	bulk, 0% crystallinity
7, Knipp04	TFT	polycrystalline	SiN_3	0.2-0.55	grain size 3-7 μm
8		polycrystalline	thermal SiO_2+OTS	0.5-1.4	grain size 1-2 μm
9, Fritz05	TFT	polycrystalline	SiO_2 rough	0.02	
10			SiO_2 smooth	0.31	
11			SiO_2+polymer	0.62	
12, Duffy08	TFT	polycrystalline	SiO_2+BCB	0.4-0.7	large crystals
13, Klauk02	TFT	polycrystalline	SiO_2	0.4	
14			SiO_2+OTS	1.0	
15			Si+polymer	3.0	
16, this work	comp.	nanocrystalline		0.2	bulk, 30% crystallinity
17, this work	comp.	nanocrystalline		0.9	bulk, 60% crystallinity
18, this work	comp.	nanocrystalline		1.8	bulk, 80% crystallinity
19, Zhang16	OFET	2D single crystal	boronitride	1.6	monolayer (1L)
20, this work	comp.	2D single crystal		4.2	monolayer (1L)
21, Zhang16	OFET	2D single crystal	boronitride	3	bilayer (2L)
22, this work	comp.	2D single crystal		7.3	bilayer (2L)
23, Lee06	OFET	single crystal	SiO_2	2.3	largest, polymorph unknown
24,				0.66	smallest, polymorph unknown
25, Takeyama12	OFET	single crystal	Al_2O_3+ionic liquid	5	polymorph I, crystal size 200 μm
26, Arabi16	OFET	single crystal	SiO_2	5.6	crystal size 50 μm
27, this work	comp.	single crystal		9.6	bulk, polymorph I

References

[1] R. B. Campbell, J. M. Robertson, J. Trotter, *Acta Crystallographica* **1961**, *14*, 7 705.

[2] Y. Zhang, J. Qiao, S. Gao, F. Hu, D. He, B. Wu, Z. Yang, B. Xu, Y. Li, Y. Shi, W. Ji, P. Wang, X. Wang, M. Xiao, H. Xu, J.-B. Xu, X. Wang, *Phys. Rev. Lett.* **2016**, *116* 016602.
